# Excess Vitamins or Imbalance of Folic Acid and Choline in the Gestational Diet Alter the Gut Microbiota and Obesogenic Effects in Wistar Rat Offspring

**DOI:** 10.3390/nu13124510

**Published:** 2021-12-16

**Authors:** Ulrik N. Mjaaseth, Jackson C. Norris, Niklas D. J. Aardema, Madison L. Bunnell, Robert E. Ward, Korry J. Hintze, Clara E. Cho

**Affiliations:** 1 Department of Nutrition, Dietetics and Food Sciences, Utah State University, Logan, UT 84322, USA; unmjaaseth@ucdavis.edu (U.N.M.); jackson.norris@aggiemail.usu.edu (J.C.N.); naardema93@gmail.com (N.D.J.A.); maddielbunnell@gmail.com (M.L.B.); robert.ward@usu.edu (R.E.W.); korry.hintze@usu.edu (K.J.H.); 2 Department of Human Health and Nutritional Sciences, University of Guelph, Guelph, ON N1G 2W1, Canada

**Keywords:** folic acid, choline, gestational nutrition, gut microbiota, obesity

## Abstract

Excess vitamin intake during pregnancy leads to obesogenic phenotypes, and folic acid accounts for many of these effects in male, but not in female, offspring. These outcomes may be modulated by another methyl nutrient choline and attributed to the gut microbiota. Pregnant Wistar rats were fed an AIN-93G diet with recommended vitamin (RV), high 10-fold multivitamin (HV), high 10-fold folic acid with recommended choline (HFol) or high 10-fold folic acid without choline (HFol-C) content. Male and female offspring were weaned to a high-fat RV diet for 12 weeks post-weaning. Removing choline from the HFol gestational diet resulted in obesogenic phenotypes that resembled more closely to HV in male and female offspring with higher body weight, food intake, glucose response to a glucose load and body fat percentage with altered activity, concentrations of short-chain fatty acids and gut microbiota composition. Gestational diet and sex of the offspring predicted the gut microbiota differences. Differentially abundant microbes may be important contributors to obesogenic outcomes across diet and sex. In conclusion, a gestational diet high in vitamins or imbalanced folic acid and choline content contributes to the gut microbiota alterations consistent with the obesogenic phenotypes of in male and female offspring.

## 1. Introduction

Inadequate or excess nutrient intake during pregnancy predisposes offspring to a greater risk of chronic diseases including obesity, type 2 diabetes, metabolic syndrome, cardiovascular disease and non-alcoholic fatty liver disease [1,2,3]. In the current pattern of over-consumption of nutrients, 77% of pregnant women are reported to use one or more dietary supplements contributing to vitamin intake levels above the recommendation [4]. Among the vitamins, folic acid is consumed in excess due to increased supplement use [5] and discretionary folic acid fortification [6], whereby 33% of pregnant women or 47% of supplement users exceed the Tolerable Upper Intake Level of 1000 µg/d for folic acid [7]. We have previously shown that feeding a high, non-toxic multivitamin (10-fold the recommended amount of multivitamins) AIN-93G diet during pregnancy produces obesogenic phenotypes in male [8] and female [9] Wistar rat offspring. We identified folic acid as a vitamin candidate responsible for greater food intake, body weight, characteristics of metabolic syndrome and changes in hypothalamic feeding-related neuropeptide expression through DNA methylation in male offspring [10]. In contrast, female offspring born to dams fed a high folic acid diet had lower body weight [11], making unclear whether another nutrient factor modifies the effect of excess folic acid on metabolic disease risk.

Choline is a bioactive micronutrient that is critical for biological functions including cellular methylation reactions, cholinergic neurotransmission, membrane biosynthesis and lipid transport and metabolism as a precursor of betaine, acetylcholine, phosphatidylcholine and sphingomyelin [12]. In contrast to folic acid, the majority of the adult population (about 90%) does not achieve the Adequate Intake (AI) for choline, including most pregnant women [13] with intakes well below the AI of 450 mg/day [14]. Further, choline is absent in most prenatal dietary supplements. However, the effects of imbalances in folic acid and choline intakes during pregnancy on development of metabolic disease risk have received limited attention.

The metabolic pathways of folate and choline are intricately linked as shown by altered folate turnover and availability of methyl groups in choline depletion/repletion studies [15,16,17], suggesting the involvement of choline in folate-related outcomes in the offspring. Moreover, folate and choline are produced and utilized by gut microbes but are also suggested to be important modifiers of the gut microbiota. Both the deficiency and supplementation of micronutrients including methyl nutrients have been shown to influence gut microbial communities such as *Clostridiales*, *Faecalibacterium*, *Akkermansia*, *Alistipes* and *Odoribacter* with consequences on host physiology [18,19,20]. Imbalances in the gut microbiota, termed gut dysbiosis, are of interest because of their emerging role as determinants of obesity, metabolic syndrome and other chronic disease risk [21]. The functional importance of the gut microbiota has also been highlighted by the modulation of host metabolic health via gut microbial-derived metabolites including short-chain fatty acids (SCFAs) [22].

As the gut microbiota may be another potential target central to shaping long-term health of the offspring, we sought to examine the offspring gut microbiota and physiologic phenotypes in the context of gestational over-consumption of vitamins or imbalances in methyl nutrients. Among many factors that may influence the offspring gut microbiota, gestational diet is being recognized to impact the initial establishment of the gut microbiota [23]. Further, recent evidence suggests that maternal-fetal transmission of microbiota during early life (*in utero* and lactation) may extend beyond infancy and persist into adulthood [23]. The offspring gut microbiota may be similar to that of maternal patterns [24], and this study focused on the offspring outcomes with an overall goal of identifying micronutrient contributors to the obesogenic phenotypes and associated gut microbiota characteristics in males and females.

We hypothesized that feeding a diet high in vitamins or imbalanced folic acid and choline content during pregnancy shifts the gut microbiota composition consistent with the obesogenic phenotypes of offspring. Our primary objective was to determine the effects of excess vitamins, folic acid or imbalanced folic acid and choline in the gestational diet on concentrations of SCFAs, gut microbiota composition and physiologic phenotypes including body weight, food intake, activity, energy expenditure, glucose response and body composition in male and female offspring. An imbalanced folic acid and choline diet consisted of a high folic acid gestational diet without choline to prevent choline from being a potential factor obscuring the effect of folic acid. Adverse birth outcomes were not expected with the absence of choline in the background of high folic acid content, supported by previous reports demonstrating that folate-related neural tube defect pathogenesis was not observed with choline deficiency [25].

## 2. Materials and Methods 

### 2.1. Animals and Diets 

First-time pregnant Wistar rats (2–4 days of pregnancy) were purchased from Charles River Laboratories (Wilmington, MA, USA). The dams were housed individually in ventilated plastic transparent cages with bedding in a 12:12 h light-dark cycle with *ad libitum* access to food and water throughout the study period. From the fourth day of pregnancy to term, dams were fed the AIN-93G diet [26] containing either the recommended vitamin (RV), high multivitamin (HV; 10-fold the recommended multivitamin mix), high folic acid (HFol; 10-fold folic acid with recommended choline) or high folic acid without choline (HFol-C; 10-fold folic acid without choline) content (Table 1; *n* = 10–12/diet group; Research Diets, New Brunswick, NJ, USA). The RV, HV, HFol and HFol-C diets were isocaloric (4.0 kcal/g). Litter size was culled to 10 within 24 h of delivery to minimize the difference in milk availability. All dams were fed the RV diet during lactation. At weaning, one male and one female offspring from each litter were fed a high-fat AIN-93G RV diet, 60 kcal% fat, from mostly lard, balanced for micronutrient-to-energy content (D12451; Research Diets, New Brunswick, NJ, USA) with the following composition (in g/kg): 239.5 lard, 123.8 maltodextrin, 200 casein, 68 sucrose, 25 soybean oil, 50 cellulose, 10 vitamin mix V10037, 35 mineral mix S10022G, 3 L-cystine and 2.5 choline bitartrate. The protocols were approved (#10113) by the Utah State University Institutional Animal Care and Use Committee.

### 2.2. Body Weight and Food Intake 

**Figure 1:**
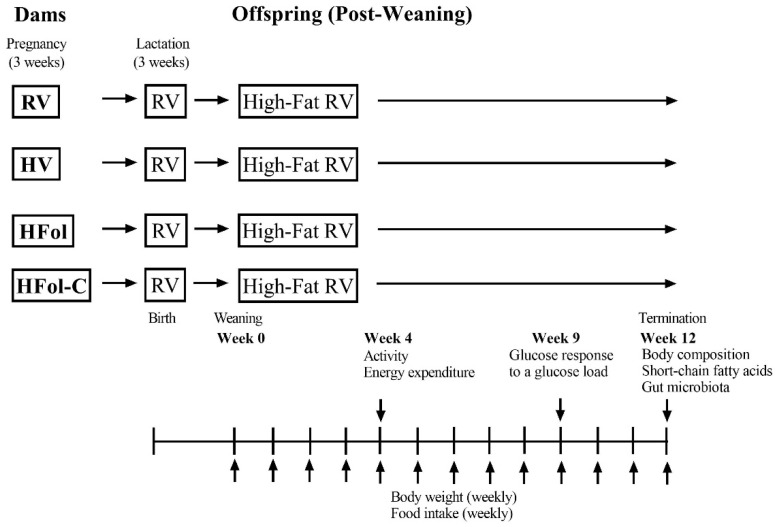
A schematic of the study timeline in male and female offspring from 0–12 weeks post-weaning. Diet abbreviations: RV, recommended vitamin; HV, high multivitamin; HFol, high folic acid with recommended choline; HFol-C, high folic acid without choline.

The study timeline is shown in Figure 1. Body weight and food intake were measured every week from 0–12 weeks of post-weaning in male and female offspring. Body weight at birth and weaning was also measured. Feed efficiency was calculated as cumulative body weight gain over 12 weeks post-weaning/energy intake (g/kcal).

### 2.3. Activity and Energy Expenditure 

At 4 weeks post-weaning, activity (locomotion and rearing) and heat production as an indicator of energy expenditure were measured in a random subset of male and female offspring (*n* = 3/diet group/sex) using the Comprehensive Lab Animal Monitoring System with a home cage implementation (CLAMS-HC; Columbus Instruments, Columbus, OH, USA). After animals were allowed to acclimatize for 12 h, ambulatory activity was measured for the X and Y axes as the number of times different infrared beams were broken during an interval of 10 min for the duration of 24 h. Any repetitive break of the same beam was included in the ambulatory count. Total rearing activity was also measured for the Z axis as the total number of times infrared beams were broken. Based on the rate of oxygen consumption (VO2) and carbon dioxide production (VCO2) from gas sensors, heat production was calculated as [3.815 + 1.232(VCO2/VO2)] × VO2. Measurements were grouped into the light and dark cycles within the CLAMS data eXamination Tool (CLAX; Columbus Instruments, Columbus, OH, USA) and those from the dark cycle were used as they represent the typical active phase.

### 2.4. Glucose Response to a Glucose Load 

At 9 weeks post-weaning, the offspring underwent a 10-h overnight fast, and baseline glucose was measured from the tail vein with a glucose meter (Precision Xtra, Abbott Laboratories, Abbott Park, IL, USA). The offspring were then administered with a glucose load (0.375 g glucose mL^–1^, 5 g of glucose per kg body weight) via oral gavage. Subsequent blood glucose concentrations were measured at 15, 30, 45 and 60 min and the incremental area under the curve (iAUC) was calculated.

### 2.5. Body Composition and Fecal Sample Collection

At 12 weeks post-weaning, body composition was measured by magnetic resonance imaging (MRI) scan (EchoMRI-700; EchoMRI, Houston, TX, USA). Body fat percentage was calculated as (fat mass/total mass) × 100. The rats were terminated at 12 weeks post-weaning via decapitation. Fecal samples from the offspring were collected directly from the colon at necropsy and frozen at –80 °C until further analyses.

### 2.6. Short-Chain Fatty Acid Analysis 

The volatile acid content of the fecal samples was measured according to the method of Ward et al. with modifications [27]. Upon sample mass determination, distilled water and a metaphosphoric acid solution (250 g/L) containing 1 g/L of ethylbutyric acid as an internal standard were added according to the ratio of 1:9:2 (sample:water:acid/internal standard). Microcentrifuge tubes were vortexed for 5 min and then centrifuged at 10,000× *g* for 10 min. Then, 200 ul of the supernatant was transferred to an insert in a gas chromatography vial. An eight point calibration was performed with a mix of seven acids. Acetic, propionic and butyric acids were present from 10 to 0.08 mM, and isobutyric, isovaleric, valeric and caproic acids were present from 5 to 0.04 mM. To prepare calibrations, 1 mL of standard was mixed with 0.2 mL of the metaphosphoric/ethylbutyric acid solution.

Samples were analyzed by gas chromatography on an Agilent 6890 gas chromatograph (equipped with a ZB-FFAP column (30 m × 0.52 mm ID × 1.0 µm film thickness; Phenomenex, Torrance, CA, USA) and a flame ionization detector. The injector temperature was at 200 °C, and 1 µL was injected at a 10:1 split ratio. The initial column temperature was 60 °C and was held for 1 min. The column was heated at 17 °C/min to 260 °C and held for 4 min. Peaks were identified according to retention time of individually run acids. For both external standards and the samples, raw peak areas were normalized to the ethylbutyric acid area. Peak area ratios were converted to concentration using regression equations for each acid. The overall concentration of acid in the sample was determined by multiplying the volume of water added by the concentration derived from the regression equation and dividing by the sample mass.

### 2.7. 16S rRNA Gene Sequencing

The experimental procedures for genomic DNA extraction and 16S rRNA sequencing were followed as previously described in [28]. Briefly, genomic DNA was extracted from 250 mg of fecal sample using the DNeasy PowerSoil Kit (Qiagen, Hilden, Germany) according to the manufacturer’s instructions. The concentration of DNA for each sample was determined using the NanoQuant spectrophotometer (Tecan, Männedorf, Switzerland) and normalized to 10 ng/µL. PCR amplification and sequencing of the V4 region of the 16S rRNA gene were performed using the 16S primers fused to the Illumina sequencing adaptor sequences 515F 5′-*ACACTCTTTCCCTACACGACGCTCTTCCGATCT* GTGYCAGCMGCCGCGGTAA-3′ and 806R 5′-*GTGACTGGAGTTCAGACGTGTGCTC TTCCGATCT*GGACTACNVGGGTWTCTAAT-3′, whereby the Illumina sequences are indicated by italics and the bacterial primers 515F and 806R are indicated by underline. A subsequent PCR reaction was performed with the indexing primers [29] using the dual-index methodology [30]. The presence and approximate size of the amplicons were confirmed with gel electrophoresis. PCR products were purified with Agencourt AMPure microbeads (Beckman Coulter, Indianapolis, IN, USA) and quantified using the Quant-iT PicoGreen dsDNA Assay Kit (Thermo Fisher Scientific, Waltham, MA, USA). Purified samples were normalized to 1 ng/µL, pooled and stored at –20 °C. Sequencing was performed at the Center for Integrated Biosystems at Utah State University using the Illumina MiSeq platform with a V2 500 cycle kit for 2 × 250 bp paired-end reads.

The 16S sequencing data were processed and analyzed using the Quantitative Insights Into Microbial Ecology (QIIME2) version 2019.10 as previously described [28]. Demultiplexed paired-end reads were quality-filtered, trimmed and denoised using the DADA2 package [31]. Taxonomy was assigned using the sklearn classifier trained on the 515F/806R region of the Silva 138 reference database [32] clustered at 99% similarity. Sequences were aligned using the MAFFT plugin [33] followed by FastTree and midpoint-root to generate a phylogenetic tree. The q2-diversity plugin was used to conduct core metric analyses with rarefication to the sample with the fewest sequences. Alpha diversity was assessed by Faith’s phylogenetic and Shannon’s diversity indices. Beta diversity was assessed by unweighted UniFrac distances and a principal coordinates analysis (PCoA) plot [34] was visualized in Emperor [35].

### 2.8. Statistical Analyses 

SAS Version 9.3 (SAS Institute Inc, Cary, NC, USA) was used to analyze the measures of phenotypes, functional outcomes and metabolism. Treatment effects on body weight and food intake were determined using one-way repeated measures analysis of variance (ANOVA) by the PROC MIXED model procedure followed by the Tukey–Kramer post hoc test, with gestational diet and time as the main factors. Feed efficiency, activity, energy expenditure, glucose response to a glucose load, body composition and concentrations of SCFAs were compared using one-way ANOVA followed by the Tukey–Kramer post hoc test.

For the gut microbiome analyses, alpha diversity measures were assessed with the Kruskal–Wallis test. Beta diversity unweighted UniFrac distance measures were tested using permutational multivariate analysis of variance (PERMANOVA) with 999 permutations via the adonis function. Log-fold differentials were generated using the Songbird plugin [36], whereby the formula included diet and sex with RV-valued samples as a reference to learn the differentials. The fitted model was compared to a baseline model to determine a pseudo-Q2 value as an indicator of predictive accuracy on the cross validation samples. Differential log-ratios of high and low ranked features (top to bottom 10% of features) were visualized using Qurro [37]. Multiple testing was corrected at 5% by the Benjamini–Hochberg False Discovery Rate method. Statistical significance was deemed at *p* ≤ 0.05. Values are reported as mean ± standard error of means (SEM).

## 3. Results

### 3.1. Body Weight and Food Intake

**Figure 2:**
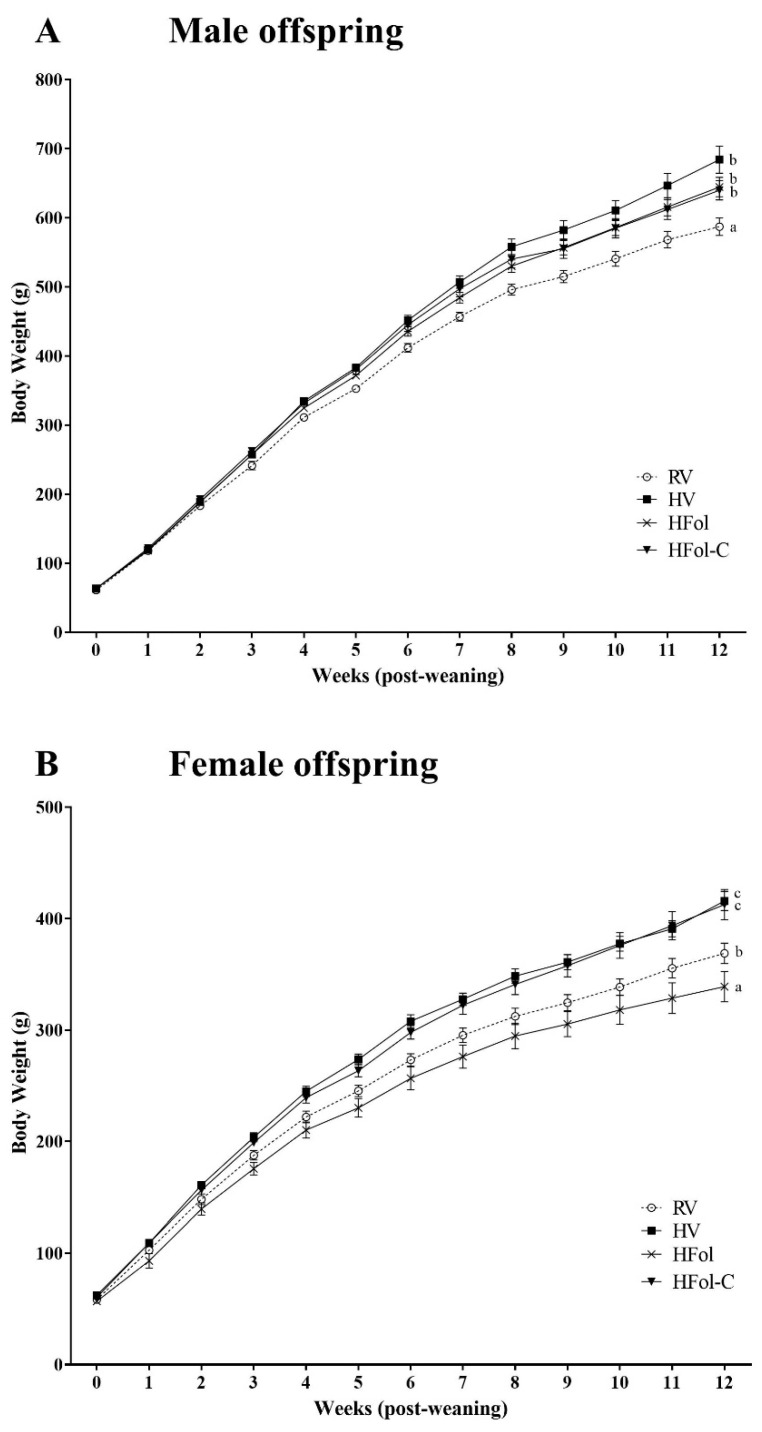
Body weight, in grams, in (**A**) male and (**B**) female offspring from 0-12 weeks post-weaning. Diet abbreviations: RV, recommended vitamin; HV, high multivitamin; HFol, high folic acid with recommended choline; HFol-C, high folic acid without choline. (**A**) Diet *p* = 0.0002, Time *p* < 0.0001, Diet ∗ Time *p* < 0.0001. (**B**) Diet *p* < 0.0001, Time *p* < 0.0001, Diet ∗ Time *p* < 0.0001. Superscripts indicate significant difference by PROC MIXED model repeated measures followed by the Tukey–Kramer post hoc test, *n* = 10–12/group. Values are mean ± SEM.

**Figure 3:**
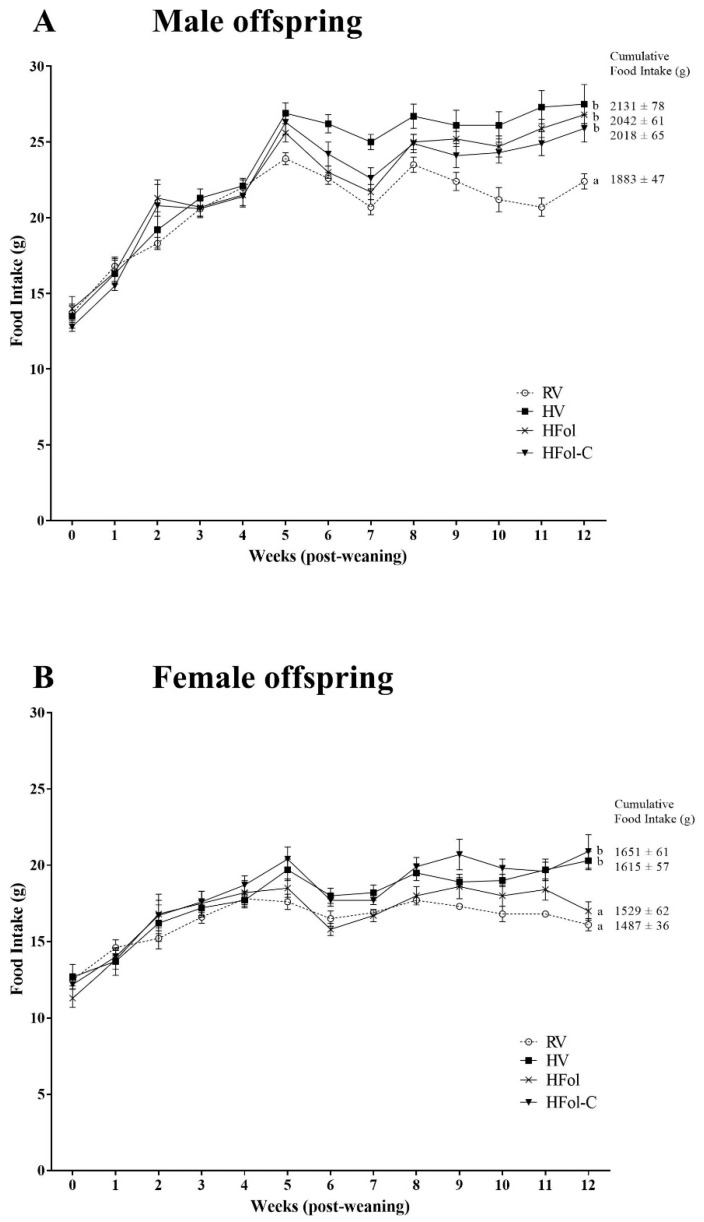
Food intake, in grams, in (**A**) male and (**B**) female offspring from 0-12 weeks post-weaning. Diet abbreviations: RV, recommended vitamin; HV, high multivitamin; HFol, high folic acid with recommended choline; HFol-C, high folic acid without choline. (**A**) Diet *p* < 0.0001, Time *p* < 0.0001, Diet ∗ Time *p* = 0.002. (**B**) Diet *p* = 0.001, Time *p* < 0.0001, Diet ∗ Time *p* = 0.04. Superscripts indicate significant difference by PROC MIXED model repeated measures followed by the Tukey–Kramer post hoc test, *n* = 10–12/group. Values are mean ± SEM.

In male offspring, the HV, HFol and HFol-C groups had 12% higher body weight than the RV group (Diet: *p* = 0.0002, Time: *p* < 0.0001, Diet ∗ Time Interaction: *p* < 0.0001; Figure 2A). In female offspring, the HFol group had 8% lower body weight, whereas the HV and the HFol-C groups had 12% higher body weight than the RV group (Diet: *p* < 0.0001, Time: *p* < 0.0001, Diet ∗ Time Interaction: *p* < 0.0001; Figure 2B). Food intake differences mirrored mostly that of body weight in both male and female offspring. In males, HV, HFol and HFol-C offspring had 10% higher food intake compared to RV offspring (Diet: *p* < 0.0001, Time: *p* < 0.0001, Diet ∗ Time Interaction: *p* = 0.002; Figure 3A). In females, HV and HFol-C offspring had 10% higher food intake compared to RV offspring (Diet: *p* = 0.001, Time: *p* < 0.0001, Diet ∗ Time Interaction: *p* = 0.04; Figure 3B) but food intake of HFol and RV offspring did not differ. No differences in body weight at birth and weaning were observed. Feed efficiency (g/kcal) for males (RV: 0.50 ± 0.01; HV: 0.52 ± 0.01; HFol: 0.50 ± 0.01; HFol-C: 0.51 ± 0.01; *p* = 0.2) and females (RV: 0.39 ± 0.01; HV: 0.40 ± 0.01; HFol: 0.35 ± 0.02; HFol-C: 0.39 ± 0.01; *p* = 0.07) did not differ among the diet groups.

### 3.2. Activity and Energy Expenditure

**Figure 4:**
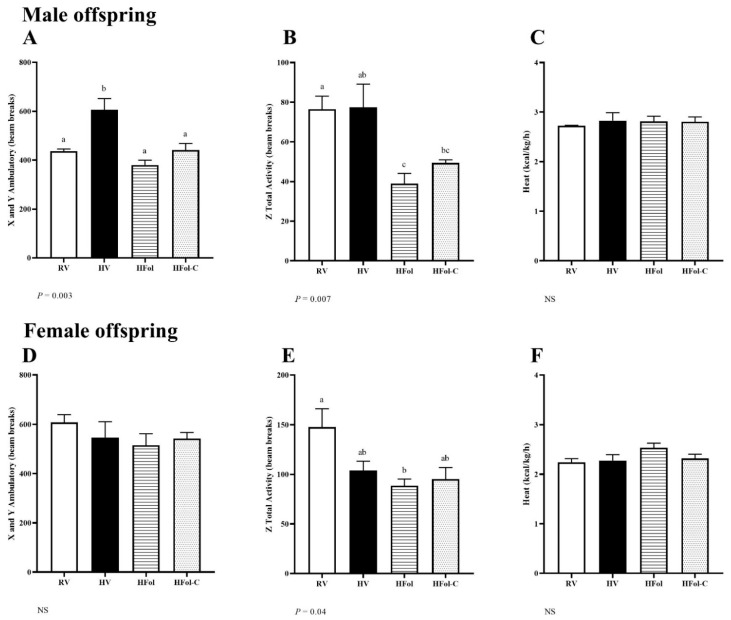
Ambulatory activity (number of beam breaks), rearing activity (total number of beam breaks) and energy expenditure (kcal/kg/h) in (**A**–**C**) male and (**D**–**F**) female offspring, respectively, at 4 weeks post-weaning. Diet abbreviations: RV, recommended vitamin; HV, high multivitamin; HFol, high folic acid with recommended choline; HFol-C, high folic acid without choline. Superscripts indicate *p* ≤ 0.05 by one-way ANOVA followed by the Tukey–Kramer post hoc test, *n* = 3/group/sex. NS denotes not significant. Values are mean ± SEM.

Indirect calorimetry was utilized to measure activity and energy expenditure to provide a comprehensive understanding of energy regulation. In male offspring, ambulatory activity was 39% higher in HV (*p* = 0.003; Figure 4A) whereas total rearing activity was 53% lower in both HFol and HFol-C (*p* = 0.007; Figure 4B) compared to in RV. In female offspring, total rearing activity was 40% lower in HFol compared to RV (*p* = 0.04; Figure 4E). No other differences in ambulatory and total activity scores were detected, nor differences in heat production as an indicator of energy expenditure.

### 3.3. Glucose Response to a Glucose Load

**Figure 5:**
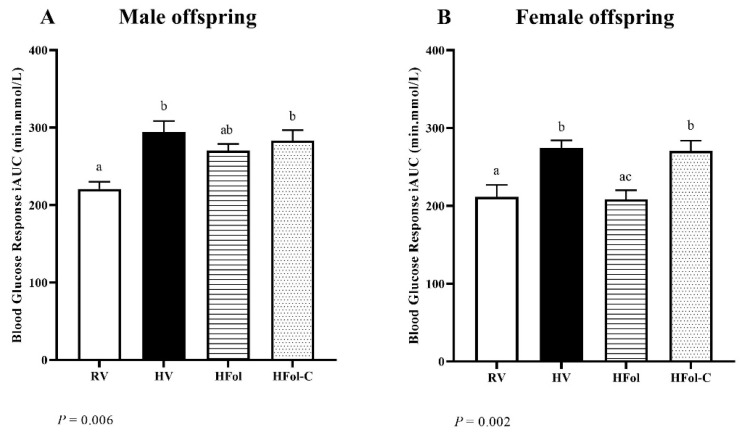
Blood glucose response to a glucose load (5 g of glucose per kg of body weight) as incremental area under the curve (iAUC) in (**A**) male and (**B**) female offspring at 9 weeks post-weaning. Diet abbreviations: RV, recommended vitamin; HV, high multivitamin; HFol, high folic acid with recommended choline; HFol-C, high folic acid without choline. Superscripts indicate *p* ≤ 0.05 by one-way ANOVA followed by the Tukey–Kramer post hoc test, *n* = 10–12/group. Values are mean ± SEM.

HV and HFol-C male offspring had a 31% higher glucose response to a glucose load compared to RV offspring (*p* = 0.006; Figure 5A). Although no difference was found between HFol and RV male offspring, HFol also did not differ from the HV nor HFol-C groups. HV and HFol-C female offspring had a 29% higher glucose response to a glucose load than RV offspring (*p* = 0.002; Figure 5B). The HFol and RV groups did not differ, but the glucose response to a glucose load was 24% lower in HFol compared to HV and HFol-C offspring.

### 3.4. Body Composition

**Figure 6:**
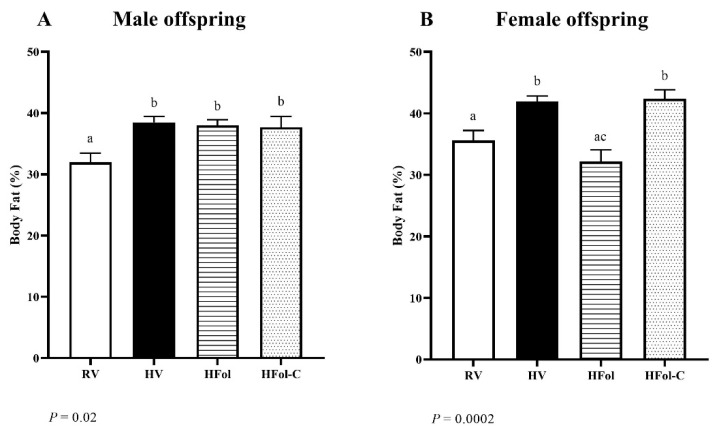
Body fat percentage, in percent, calculated as percent fat mass adjusted by total mass, in (**A**) male and (**B**) female offspring at 12 weeks post-weaning. Diet abbreviations: RV, recommended vitamin; HV, high multivitamin; HFol, high folic acid with recommended choline; HFol-C, high folic acid without choline. Superscripts indicate *p* ≤ 0.05 by one-way ANOVA followed by the Tukey–Kramer post hoc test, *n* = 10–12/group. Values are mean ± SEM.

In males, HV, HFol and HFol-C offspring had 19% higher body fat percentage compared to RV offspring (*p* = 0.02; Figure 6A). In females, HV and HFol-C offspring had 18% higher body fat percentage compared to RV offspring (*p* = 0.0002; Figure 6B). HFol and RV female offspring did not differ, but HFol female offspring had 24% lower body fat percentage than HV and HFol-C offspring.

### 3.5. Short-Chain Fatty Acid Concentrations

In male offspring, the butyric acid concentration was 35% lower in the HFol and HFol-C groups compared to RV (*p* = 0.0004; Table 2A) without any difference between HV and RV. In female offspring, the acetic acid concentration was 30% higher in the HV and HFol-C groups (*p* < 0.0001; Table 2B), whereas HFol did not differ compared to RV but was 32% lower compared to HV and HFol-C. Other SCFAs did not differ among the diet groups.

### 3.6. Gut Microbiota Composition

**Figure 7:**
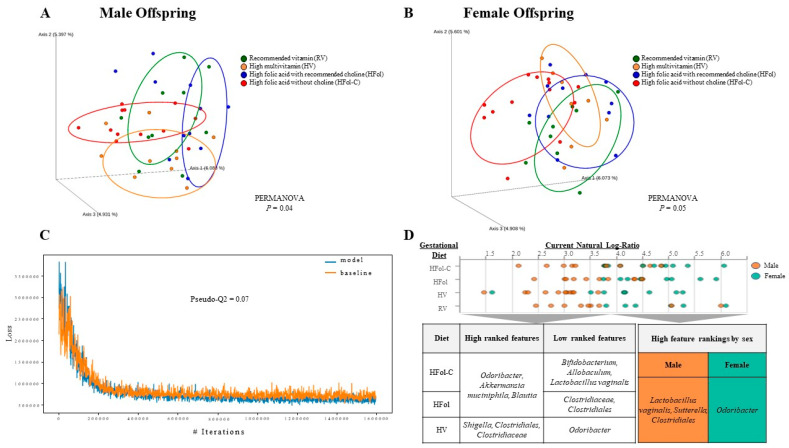
A principal coordinates analysis (PCoA) of the unweighted UniFrac distances in (**A**) male and (**B**) female offspring at 12 weeks post-weaning. Diet abbreviations: RV, recommended vitamin; HV, high multivitamin; HFol, high folic acid with recommended choline; HFol-C, high folic acid without choline. Gut microbiota composition differed among the diet groups in male (*p* = 0.04, *n* = 10–11/group) and female (*p* = 0.05, *n* = 10–11/group) offspring by PERMANOVA with 999 permutations via the adonis function. (**C**) Comparison of the Songbird multinomial regression model with diet and sex as covariates against a baseline model (pseudo-Q2 value = 0.07). (**D**) Differential log-ratios explored as high and low ranked features (top to bottom 10% of features) associated with diet and sex as visualized with Qurro.

The overall gut microbial communities among the diet groups differed in both male (PERMANOVA *p* = 0.04 with 999 permutations via the adonis function; Figure 7A) and female (PERMANOVA *p* = 0.05 with 999 permutations via the adonis function; Figure 7B) offspring as assessed by unweighted UniFrac distances. No difference in alpha diversity measures was detected for Faith’s phylogenetic and Shannon’s diversity indices (data not shown). Differential taxonomic abundance analysis was then conducted using Songbird multinomial regression. Comparison of the Songbird model to a baseline model with diet and sex as covariates achieved a pseudo-Q2 value of 0.07 (Figure 7C), but using diet alone separated by sex of the offspring as a covariate resulted in overfitting of the multinomial regression model with a pseudo-Q2 value < 0. Differential log-ratios were then explored as high and low ranked features (top to bottom 10% of features) associated with diet and sex (Figure 7D). Compared to RV, high ranked features for HV included *Shigella*, *Clostridiales*, *Clostridiaceae* and low ranked features included *Odoribacter*. For HFol and HFol-C, high ranked features consisted of *Odoribacter*, *Akkermansia muciniphila* and *Blautia*, but these two diet groups differed in low ranked features. For HFol-C, low ranked features included *Bifidobacterium*, *Allobaculum* and *Lactobacillus vaginalis*. For HFol, low ranked features included *Clostridiaceae* and *Clostridiales*. In males, high ranked features were represented by *Lactobacillus vaginalis*, *Sutterella* and *Clostridiales*, whereas in females, high ranked features included *Odoribacter*.

## 4. Discussion

The findings from this study support our hypothesis that feeding a diet high in vitamins or imbalanced in folic acid and choline content during pregnancy shifts the gut microbiota consistent with the obesogenic phenotypes of offspring. Removing choline eliminated the contrasting responses to a high folic acid gestational diet in male and female offspring, suggesting an important role of choline in conjunction with folic acid in the modification of obesity risk. We demonstrate for the first time that the concentrations of SCFAs differed among the diet groups in male and female offspring, indicating metabolic consequences arising from the gut microbial communities. Both the gestational diet and sex of the offspring were determinants of the gut microbiota composition, which may shape the risk of metabolic diseases in adulthood.

A diet high in vitamins or an imbalance of folic acid and choline contributed to higher body weight, food intake, glucose response and body fat in male offspring. Consistent with the previous findings [8,9,10,38], male offspring of dams fed the HV diet displayed characteristics of metabolic syndrome, and folic acid accounts for many of these outcomes, as observed [39] with higher body weight, food intake and body fat percentage, thus serving as a benchmark of reproducible outcomes. However, folic acid in the gestational diet had a minor role in glucose regulation as male offspring of HFol dams did not differ in glucose response to a glucose load compared to all other diet groups. Removing choline from the HFol diet was novel in showing higher body weight, food intake, glucose response to a glucose load and body fat percentage compared to the RV group, suggesting the importance of folic acid and choline imbalance as a contributor to the obesogenic outcomes. Our findings are similar to another report showing that a diet containing 5-fold folic acid and 0.5-fold choline fed during pregnancy led to greater weight gain and food intake in periadolescent male offspring [40]. However, in this report, the obesogenic effects became less apparent in adulthood, which may be due to the smaller imbalance between folic acid and choline content compared to our current study design.

In contrast to male offspring of dams fed the HFol diet exhibiting the obesogenic phenotypes, female offspring had lower body weight without differences in food intake as observed previously [11], as well as glucose response to a glucose load and body fat percentage that did not differ compared to the control offspring. Feed efficiency appeared lower in the female offspring of HFol dams but did not reach statistical significance, which suggests that other aspects of energy balance metabolism may explain reduced body weight. Sex-specific differences have been found in other models of obesity that propose more resistance to the adverse effects of altered metabolism in females compared to males [41,42]. When choline was removed, we revealed that the contrasting outcomes of the HFol gestational diet in female offspring were eliminated. An imbalance of folic acid and choline produced female offspring with higher body weight, food intake, glucose response and body fat percentage than the control group, and these effects did not differ compared to the HV females, suggesting that choline was an important factor that modified the obesogenic effects of a HFol gestational diet. Overall, the micronutrient composition of the gestational diet shaped health trajectories that differed by the sex of the offspring.

Disturbances in energy balance due to high vitamins or imbalanced folic acid and choline content were shown as altered activity in male and female offspring. We utilized CLAMS-HC to determine activity and energy expenditure during the dark phase, which is typically the most active time for rodents. Male offspring born to dams fed the HV gestational diet had higher ambulatory activity scores compared to the control offspring. It is suggested that voluntary activity counteracts the effect of hyperphagia [43], but HV male offspring exhibited greater body weight and body fat percentage in conjunction with higher food intake patterns, indicating that increased activity did not appear to correct metabolic disruptions associated with obesity in these offspring. Male offspring of dams fed the HFol or HFol-C gestational diet had lower rearing activity, which reduced physical activity and higher food intake contributed to positive energy balance. A past investigations using low choline showed lower activity and energy expenditure in male offspring [44], which suggests that lack of choline itself may program adverse long-term phenotypes. In females, the HV, HFol and HFol-C groups all showed reduced rearing activity with HFol being lower than the control, and HV and HFol-C that did not differ from the HFol and RV offspring. The reduction in rearing activity reflected the obesogenic phenotypes of HV and HFol-C females but not in HFol females that had lower body weight and no difference in body fat percentage when compared to the control offspring. Energy expenditure did not differ in male and female offspring even though patterns of activity were dysregulated and may be governed by different efficiencies in nutrient absorption which were not captured with the food intake, activity or body composition measures.

We demonstrated for the first time that the micronutrient composition of the gestational diet modifies the metabolic activity of the gut microbiota in male and female offspring. Male offspring from the HFol and HFol-C groups had lower concentrations of butyric acid, a SCFA that has been found to provide a protective role against obesity and related metabolic diseases [45], and this reduced protection was consistent with the obesogenic phenotypes including greater weight gain, food intake, body fat and glucose response. Excess folic acid rather than the choline content may be linked to reduced butyric acid concentrations as the HFol and HFol-C males did not differ, and that another mechanism other than SCFA may underlie the obesogenic phenotypes of HV offspring. In females, both the HV and HFol-C offspring that displayed the obesogenic phenotypes had greater acetic acid concentrations, whereas female offspring from the HFol group did not differ from the control offspring. The most abundant SCFA acetate has been shown to confer metabolic benefits [46], but our results are consistent with another report demonstrating a positive relationship between acetate levels and obesity [47], which partly may be a compensatory mechanism of greater energy intakes.

Differences in beta diversity measures in male and female offspring provided novel insights into the role of excess or imbalanced micronutrients consumed during pregnancy in modification of the gut microbiota composition. Our findings support recent evidence of early programming of the gut microbiota due to the maternal diet with effects on the long-term physiologic phenotypes [48]. A previous study using methyl donors including folic acid, vitamin B12, betaine and choline showed prolonged microbiome changes, although less bacterial species were detected in postnatal day 90 (P90) compared to P30 [49]. Our current study used fecal samples at post-weaning week 10 (postnatal day 91) with differences across the diet groups, although the timing of these effects are unknown as our samples were restricted to post-weaning. Previous studies have shown the importance of early life influences including those during lactation as another window of developmental changes in the gut microbiota [23]. We also do not have any direct microbiota measurements in the dams to determine maternal effects, and separate studies are warranted to relate between maternal and offspring gut microbiota patterns. Our previous investigation has revealed that dams fed a HV or methyl vitamin diet during pregnancy gained more weight 2–15 weeks post-weaning [50], suggesting that phenotypes of the dams can be modified beyond the gestational period, and may extend to other characteristics including the gut microbiota.

Differential abundance analysis was explored using the Songbird multinomial regression, whereby both diet and sex were factors that predicted accuracy of the model, but diet alone separated by sex of the offspring was not sufficient to utilize the ranked differentials. HV offspring had high ranked features of *Shigella*, *Clostridiales* and *Clostridiaceae* with low ranked features of *Odoribacter*, consistent with the previously observed inverse association between *Odoribacter* and diseases [51]. Independent of the choline content, excess folic acid in the gestational diet contributed to high ranked features of *Odoribacter*, *Akkermansia muciniphila* and *Blautia*, which high levels have previously been associated with beneficial metabolic effects [52,53,54]. Low ranked features differed whereby HFol had low ranked features of *Clostridiaceae* and *Clostridiales*, where obesity has previously been characterized by lower abundance of *Clostridiaceae* and *Clostridiales* [55]. HFol-C had low ranked features of *Bifidobacterium*, *Allobaculum* and *Lactobacillus vaginalis*, which have previously been attributed to metabolic health promoting effects [56,57,58], and may characterize the obesogenic phenotypes in male and female offspring. Differential abundance analysis also showed high ranked features of *Lactobacillus vaginalis*, *Sutterella* and *Clostridiales* in males as compared to high ranked features of *Odoribacter* in females, which may underlie sex-specific differences in the development of obesity. Thus, abundances of gut microbial populations differed by the gestational diet composition and sex of the offspring that may reflect outcome responses in males and females.

Several limitations arose in this study. Our gut microbiota measures were restricted to the post-weaning samples and thus lack elucidating any causal relationship between changes in the gut microbiota and development of the obesogenic phenotypes in the offspring. Comparison across development including changes in the maternal gut microbiota would clarify the timing of disruptions as highlighted by another report that shows maternal effects on programming of the offspring obesity risk [59]. Although energy balance disruptions in the offspring occurred as informed by the use of CLAMS-HC, our data were limited to a very small sample size (3 animals per diet per sex as limited by the number of available cages) as well as activity and energy expenditure measures at 4 weeks post-weaning when differentiation of body weight gain was not evident. Subsequent measures between 9–12 weeks post-weaning in adult offspring are needed, including those that determine energy efficiencies to identify connections among the major components of energy metabolism. Further, the absence of choline in the HFol gestational diet was meant to maximally create an imbalance; however, such an extreme imbalance may not be applicable to human consumption. Our findings using the high folic acid diet without choline illustrate that dose, nutrient composition and sex of the offspring determine the obesogenic phenotypes. Lastly, we have yet to characterize the interplay of gut microbial alterations and epigenetic mechanisms that provide a link to our previous work showing DNA methylation changes of food intake regulatory genes [10,39]. Future studies examining the availability of methyl donors due to disturbances in the gut microbiota and their SCFAs will help elucidate the early programming of metabolic disease risk.

## 5. Conclusions

Feeding a diet high in vitamins or imbalanced folic acid and choline content during pregnancy alters the gut microbiota consistent with the obesogenic phenotypes in male and female offspring. In the current pattern of widespread use of dietary supplements, discretionary fortification of folic acid and under-consumption of choline, this research highlights the importance of the amount and balance of micronutrients as factors determining health outcomes and may inform the development of public health and regulatory policies.

## Figures and Tables

**Table 1:** Composition of gestational diets.

	RV (D10012G)	HV (D18072701)	HFol (D18072702)	HFol-C (D18072703)
	g% diet (kcal%)			
Protein	20 (20)	20 (20)	20 (20)	20 (20)
Carbohydrate	64 (64)	64 (64)	64 (64)	64 (64)
Fat	7 (16)	7 (16)	7 (16)	7 (16)
Ingredient	g/kg diet (kcal/kg diet)			
Casein	200 (800)	200 (800)	200 (800)	200 (800)
L-cystine	3 (12)	3 (12)	3 (12)	3 (12)
Cornstarch	397 (1590)	397 (1590)	397 (1590)	397 (1590)
Maltodextrin 10	132 (528)	132 (528)	132 (528)	132 (528)
Sucrose	100 (400)	10 (40)	100 (400)	100 (400)
Sucrose from vitamin mix	9.75	97.5	9.75	9.75
Cellulose, BW200	50 (0)	50 (0)	50 (0)	50 (0)
Soybean oil	70 (630)	70 (630)	70 (630)	70 (630)
Tert-butylhydroquinone	0.014 (0)	0.014 (0)	0.014 (0)	0.014 (0)
Mineral mix S10022G	35 (0)	35 (0)	35 (0)	35 (0)
Vitamin mix V10037	10 (40)	100 (400)	10 (40)	10 (40)
Folic acid from vitamin mix	0.002 (0)	0.02 (0)	0.002 (0)	0.002 (0)
Folic acid added			0.018 (0)	0.018 (0)
Choline bitartrate	2.5 (0)	2.5 (0)	2.5 (0)	0 (0)
Total	1000 (4000)	1000 (4000)	1000 (4000)	998 (4000)

Pregnant dams were fed the AIN-93G diet containing either the recommended vitamin (RV), high multivitamin (HV; 10-fold multivitamin mix), high folic acid (HFol; 10-fold folic acid with recommended choline) or high folic acid without choline (HFol-C; 10-fold folic acid without choline) content.

**Table 2:** Concentrations of short-chain fatty acids (acetic acid, propionic acid, isobutyric acid, butyric acid, isovaleric acid, valeric acid and caproic acid) in µmol/g at 12 weeks post-weaning in (A) male and (B) female offspring.

(µmol/g)	Acetic Acid	Propionic Acid	Isobutyric Acid	Butyric Acid	Isovaleric Acid	Valeric Acid	Caproic Acid
**A Male offspring**							
RV	4.91 ± 0.40	0.78 ± 0.04	0.13 ± 0.01	0.82 ± 0.08 ^a^	0.12 ± 0.01	0.13 ± 0.01	0.09 ± 0.01
HV	5.05 ± 0.39	0.93 ± 0.07	0.15 ± 0.01	0.86 ± 0.07 ^a^	0.13 ± 0.01	0.15 ± 0.01	0.08 ± 0.00
HFol	4.25 ± 0.24	0.85 ± 0.03	0.13 ± 0.01	0.58 ± 0.03 ^b^	0.12 ± 0.01	0.13 ± 0.00	0.08 ± 0.00
HFol-C	4.17 ± 0.35	0.76 ± 0.06	0.13 ± 0.01	0.49 ± 0.04 ^b^	0.12 ± 0.01	0.13 ± 0.01	0.08 ± 0.00
*p* value	NS	NS	NS	*p* = 0.0004	NS	NS	NS
**B Female offspring**							
RV	4.21 ± 0.25 ^a^	0.74 ± 0.03	0.13 ± 0.01	0.69 ± 0.08	0.11 ± 0.01	0.13 ± 0.01	0.07 ± 0.00
HV	5.50 ± 0.32 ^b^	0.81 ± 0.04	0.13 ± 0.00	0.78 ± 0.09	0.11 ± 0.01	0.13 ± 0.00	0.08 ± 0.00
HFol	3.73 ± 0.16 ^a^	0.77 ± 0.07	0.11 ± 0.01	0.54 ± 0.06	0.10 ± 0.01	0.11 ± 0.01	0.07 ± 0.00
HFol-C	5.47 ± 0.19 ^b^	0.82 ± 0.06	0.13 ± 0.01	0.70 ± 0.06	0.11 ± 0.01	0.13 ± 0.01	0.07 ± 0.00
*p* value	*p* < 0.0001	NS	NS	NS	NS	NS	NS

Diet abbreviations: RV, recommended vitamin; HV, high multivitamin; HFol, high folic acid with recommended choline; HFol-C, high folic acid without choline. Superscripts indicate *p* ≤ 0.05 by one-way ANOVA followed by the Tukey–Kramer post hoc test, n = 9–12/group. NS denotes not significant. Values are mean ± SEM.
